# Insect-specific virus platforms for arbovirus vaccine development

**DOI:** 10.3389/fimmu.2025.1521104

**Published:** 2025-03-14

**Authors:** Roy A. Hall, Wilson Nguyen, Alexander A. Khromykh, Andreas Suhrbier

**Affiliations:** ^1^ School of Chemistry and Molecular Biosciences, University of Queensland, St. Lucia, QLD, Australia; ^2^ Global Virus Network Centre of Excellence, Australian Infectious Diseases Research Centre, Brisbane, QLD, Australia; ^3^ Inflammation Biology Group, QIMR Berghofer Medical Research Institute, Brisbane, QLD, Australia

**Keywords:** vaccine, arbovirus, Eilat virus, Binjari virus, Yada Yada virus, Aripo virus, YN15-283-02 virus, Chaoyang virus

## Abstract

Certain insect-specific viruses (ISVs), specifically the mosquito alphaviruses, Eilat and Yada Yada viruses, and orthoflaviviruses, Binjari, Aripo, YN15-283-02 and Chaoyang viruses, have emerged as potential platforms for generation of whole virus vaccines for human and veterinary applications. These ISVs are remarkably tolerant of the substitution of their structural polyproteins with those of alphaviruses and orthoflaviviruses that are pathogenic in humans and/or animals. The resulting ISV-based chimeric vaccines have been evaluated in mouse models and have demonstrated safety and efficacy in non-human primates, crocodiles and pigs. Targets include chikungunya, Venezuelan and eastern equine encephalitis, dengue, Zika, yellow fever, Japanese encephalitis and West Nile viruses. ISV-based chimeric vaccines provide authentically folded tertiary and quaternary whole virion particle structures to the immune system, a key feature for induction of protective antibody responses. These vaccines are manufactured in C6/36 or C7-10 mosquito cell lines, where they grow to high titers, but they do not replicate in vertebrate vaccine recipients. This review discusses the progress of these emerging technologies and addresses challenges related to adjuvanting, safety, and manufacturing.

## 1 Introduction

The WHO announced the Global Arbovirus Initiative in 2022 in response to the growing concerns over expanding outbreaks of arboviral diseases (1), which are primarily caused by pathogenic viruses in the genus *Alphavirus* (family *Togaviridae*) and the genus *Orthoflavivirus* (family *Flaviviridae*) (2). Urbanization, globalization, human mobility, and climate change, with the ensuing expansion of mosquito vectors, are all anticipated to increase the global burden of arboviral diseases (1, 3). A key intervention has been the development of vaccines (2) and herein we describe an emerging set of technologies that use mosquito alphaviruses and orthoflaviviruses to generate chimeric arboviral vaccines for human and veterinary applications.

**Figure 1:**
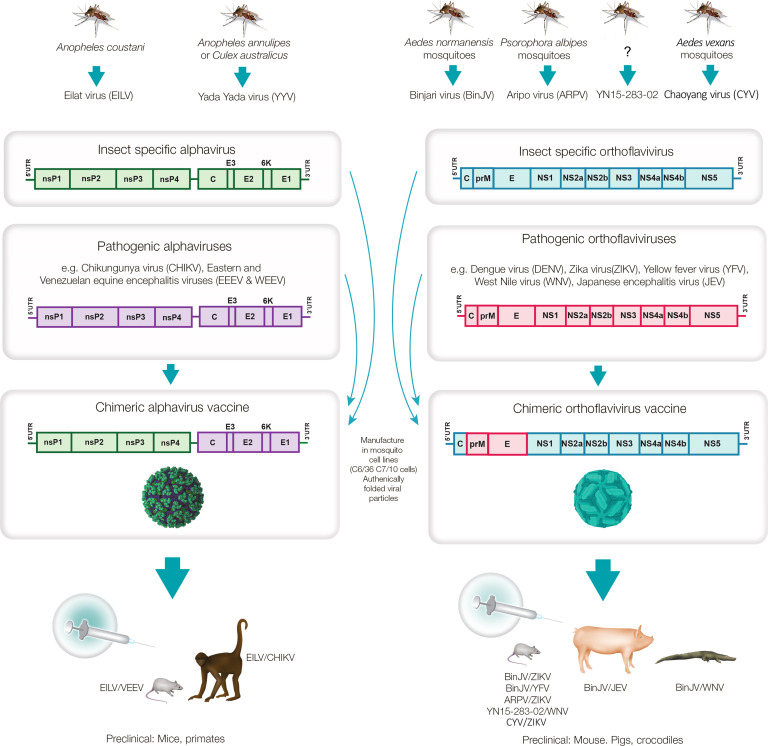
Mosquito-derived ISVs used to generate chimeric virus vaccines against pathogenic alphaviruses and orthoflaviviruses. The mosquito alphaviruses, Eilat virus and Yada Yada virus, and the mosquito orthoflaviruses, Binjari virus, Aripo virus, YN15-283-02 and Chaoyang virus have been used to generate chimeric viruses encoding the structural polyproteins of pathogenic alphaviruses (C-E3-E2-6K-E1) and orthoflaviviruses (CprME), respectively. The chimeric viruses replicate to high titers in mosquito cell lines to produce authentically folded virion particles that can be used as whole-virion vaccines that have provided protective immune responses in a number of animal species. (?) - YN15-283-02 was derived from midge (*Culicoides*) samples, but the virus was described as a mosquito orthoflavivirus.

Insect-specific viruses (ISVs) are viruses that replicate only in insects, and are distinct from arboviruses, which can replicate both in arthropod vectors (including insects) and vertebrate animal hosts. A range of factors prevent ISVs from infecting vertebrate cells, with such restriction occurring at various stages in the replication cycles (4–11). ISVs arguably represent the dark matter of virology, with vast numbers of ISVs identified by metagenomics (12–17), but only a few isolated and their behavior studied *in vivo* and *in vitro* (18). Studies so far indicate that ISVs are generally transmitted vertically from infected females to their offspring via the eggs (9, 19). Some ISVs appear to exhibit a narrow host range, with, for instance, some mosquito ISVs reported to infect only a limited number of mosquito species (12, 20, 21). ISVs are being explored as potential biological control agents for insects that threaten agricultural crops (22). Of some interest has also been the infection of mosquitoes with certain ISVs in order to inhibit, via various mechanisms, replication of pathogenic arboviruses in those mosquitoes (2, 23–26). However, herein we focus on six ISVs from mosquitoes, specifically, two alphaviruses, Eilat virus (EILV) and Yada Yada virus (YYV) and four orthoflaviviruses, Binjari virus (BinJV), Aripo virus (ARPV), YN15-283-02 virus and Chaoyang virus (CYV). For these ISVs, their structural genes can be exchanged with the structural genes from a range of pathogenic alphaviruses and orthoflaviviruses, respectively. A resulting chimeric virus would thus encode the structural proteins of a pathogenic arbovirus and the non-structural polyproteins of one of the aforementioned ISVs. These chimeric viruses are able to replicate efficiently in mosquito cell lines, but are unable to replicate in vertebrate cells. This has allowed the development of a range of ISV-based chimeric vaccines for a number of arboviral diseases (**Figure 1**).

A series of chimeric vaccines based on EILV, YYV, BinJV, ARPV, YN15-283-02 and CYV have now been described. They are manufactured in mosquito cell lines and resemble virus-like-particle (VLP) vaccines, as they essentially represent whole-virus, protein-based vaccines that cannot generate viral progeny in vertebrate vaccine recipients (**Figure 1**). They differ from VLP vaccines (27) in that they contain a fully functioning viral genome that is replication competent in mosquito cells. ISV-chimeric vaccines also differ from licensed live-attenuated orthoflavivirus chimeric virus vaccines such as Imojev, for Japanese encephalitis virus (JEV) and Dengvaxia for dengue virus (DENV), which are replication competent in vaccine recipients. The latter encode the structural proteins of JEV and DENV, but are attenuated as they encode the non-structural proteins of the yellow fever virus (YFV) 17D vaccine strain (2). An experimental chimeric live-attenuated alphavirus vaccine has also been reported for Venezuelan Equine Encephalitis virus (VEEV), and encodes the structural proteins of VEEV (TC-83 vaccine strain) and the non-structural proteins of Sindbis virus (28). Thus, chimeric vaccines for alphaviruses and orthoflaviviruses are well described; however, in contrast to the aforementioned live-attenuated chimeric vaccines, ISV-based chimeric vaccines are naturally replication-defective in vaccine recipients as the ISV RNA replication complex, encoded by the non-structural proteins, is non-functional in vertebrate cells. Like many VLP vaccines [e.g. (29, 30)], ISV-based chimeric vaccines present authentically folded structural proteins of the pathogenic arboviruses to the immune system and thereby promote induction of effective neutralizing antibody responses (2).

Herein we describe the ISV-based chimeric vaccine technologies and focus on the immunological issues associated with these technologies including potential self-adjuvanting activity, formulation with adjuvants, provision of authentic tertiary and quaternary structures, safety issues and manufacturing.

## 2 The ISV-based chimeric virus vaccine platforms

### 2.1 The Eilat virus platform for alphavirus vaccine development

Eilat virus (EILV) is an alphavirus isolated from a pool of *Anopheles coustani* mosquitoes from the Negev desert of Israel (31) and arguably represents the “poster child” for using ISV chimeras for vaccine development (32, 33). Standard cloning methodology, using an infectious cDNA clone of EILV, was used to generate the EILV chimeras with the structural polyprotein (C-E3-E2-6K-E1) of pathogenic alphaviruses replacing those from EILV to generate chimeric viruses (**Figure 1**) (32, 34). These chimeric viruses are unable to replicate in vertebrate cells even after electroporation of chimeric RNA genomes into vertebrate cell lines. Furthermore, no overt adverse outcomes were seen even after intracranial injections into *Ifnar* ^-/-^ mice (35).

A number of chimeras were generated including, Mayaro virus (EILV/MAYV), o’nyong-nyong virus (EILV/ONNV), Sindbis virus (EILV/SINV) and western equine encephalitis virus (WEEV) (32). In addition, EILV-based chimeric viruses have been evaluated as vaccines for Venezuelan equine encephalitis virus (EILV/VEEV), eastern equine encephalitis virus (EILV/EEEV) (33), and chikungunya (EILV/CHIKV) (35, 36). These vaccines, without adjuvant formulation, provided protection against challenge in mouse and non-human primate (NHP) models after a single immunization (**Figure 1**, **Table 1**, **Supplementary Table 1**).

**Table 1:** Eilat virus (EILV) chimeric vaccines for pathogenic alphaviruses evaluated in mice and NHPs.

Pathogenic alphavirus	Chimera	Animal	Dose, route of administration	Adjuvant	Readouts	Ref
Chikungunya virus (99659)	EILV/CHIKV	Mice: young C57BL/6	8.8 log_10_PFU live or formalin-inactivated s.c.	No adjuvant	Neutralizing antibodies, CD8 T cells. Protection against challenge	(35)
		Mice: adult *Ifnar* ^-/-^	8.5 log_10_PFU live or formalin-inactivated s.c.		Neutralizing antibodies. Protection against challenge.	
		Mice: C57BL/6	10^4^-10^8^ PFU i.p		IgM and IgG antibodies, memory B cell and CD8^+^ T cells	(34)
		NHPs (*Macaca fascicularis*)	1.3 x 10^6^ or 1.3 x 10^8^ PFU i.m.		Neutralizing anti-bodies, B and T cells, RNA-Seq. Adopted transfer protection in mice.	(36)
Venezuelan equine encephalitis virus	EILV/VEEV	Mice: CD-1	10^8^ PFU s.c.		Neutralizing antibodies. Protection against challenge	(33)
Eastern equine encephalitis virus	EILV/EEEV	Mice: CD-1	10^8^ PFU s.c.		Neutralizing antibodies. Protection against challenge.	

Please see **Supplementary Table 1** for details

### 2.2 The Yada Yada virus platform for alphavirus vaccine development

Yada Yada virus (YYV) was initially identified by metagenomic sequencing of mosquitoes trapped as part of the Victorian Arbovirus Disease Control Program (Australia) in 2016 (37). YYV shows a 75.7% amino acid identity with EILV (37) (GenBank QGR15363.1). Infectious YYV was generated using circular polymerase extension reaction (CPER) methodology, with transfection of C6/36 cells (38). In brief, CPER involves stitching together overlapping synthetic dsDNA fragments covering the complete viral genome, with a circularizing linker fragment containing a promoter for transcribing viral RNA in cells. Replicating virus is then recovered after direct transfection of CPER into mammalian or insect cells capable of supporting replication (8, 39–42). A simplified schematic explaining the CPER methodology is provided in **Supplementary Figure 1**.

CPER was used to generate YYV chimeric viruses, with the structural polyprotein of YYV replaced with those from pathogenic alphaviruses. The chimeras included CHIKV (YYV/CHIKV), SINV (YYV/SINV), as well as Ross River virus (YYV/RRV) and Barmah Forest virus (YYV/BFV) (**Figure 1**), with both YYV and the YYV-chimeras unable to replicate in vertebrate cell lines (38). RRV and BFV are Australasian arthritogenic alphaviruses with symptoms similar to, but usually less severe than, CHIKV (43–45). An inactivated whole virus RRV vaccine was previously shown to be well tolerated and immunogenic in a phase 3 human trial (46). The YYV/CHIKV chimera (38) is currently undergoing evaluation as a vaccine to protect against CHIKV in an adult wild-type mouse model (47).

### 2.3 The Binjari virus platform for orthoflavivirus vaccine development

Binjari virus (BinJV) was isolated in 2016 from a pool of *Aedes normanensis* mosquitoes trapped at the Bradshaw Field Training Area (Northern Territory, Australia). The sequence (GenBank; MG587038) illustrated that BinJV grouped with the lineage II insect-specific flaviviruses (5, 48). BinJV chimeric virus vaccines were generated using the CPER methodology, with the prME genes of BinJV replaced with the prME genes of pathogenic orthoflaviviruses (**Figure 1**), with both BinJV and the BinJV-chimeras unable to replicate in vertebrate cell lines (48). BinJV-based chimeric vaccines have now been generated for a range of pathogenic flaviviruses and evaluated in mouse models, crocodiles and pigs (**Table 2**; **Supplementary Table 2**).

**Table 2:** Binjari virus (BinJV) chimeric vaccines for pathogenic orthoflaviviruses evaluated in mice, crocodiles and pigs.

Pathogenic flavivirus	Chimera	Animal	Dose, route of administration	Adjuvant	Readouts	Ref
Zika virus (Natal)	BinJV/ZIKV	Mice: Male *Ifnar* ^-/-^	2 µg and 20 µg i.m.	AddaVax (InvivoGen) or unadjuvanted	ELISA and neutralizing antibody titers. Protection against challenge.	(48)
		Mice: Female *Ifnar* ^-/-^	2x 10 µg and 20 µg i.m.	AddaVax (InvivoGen) or unadjuvanted		
		Mice Pregnant *Ifnar* ^-/-^	1x 20 µg i.m.	No adjuvant	Antibodies as above. Protection against fetal brain infection & fetal abnormalities	(198)
		Mice: Female *Ifnar* ^-/-^	1x 10 µg i.m.	No adjuvant	Antibodies at 14 m and protection from challenge at 15 m.	(139)
West Nile virus (WNV_KUN_)	BinJV/WNV_KUN_	Mice: Male and female CD1	Live or UV inactivated, 2x 1 µg and 1x 5 µg s.c.	Advax (Vaxine Pty Ltd) or unadjuvanted	ELISA and neutralizing antibody titers. Protection against WNV_NY99_ challenge	(106)
Dengue virus (DENV2 D220)	BinJV/DENV2	Mice: Female AG129	1x and 2x micro-array patch. Also 3x 1 µg s.c. or i.d.	No adjuvant on patch.	ELISA and neutralizing antibody titers. Protection against challenge.	(68)
Yellow fever virus vaccine strain (YFV 17D)	BinJV/YFV_17D_	Mice: Female *Ifnar* ^-/-^	2x 5 µg, 10 µg or 20 µg of i.m.	MPLA/QS-21	Neutralizing antibodies. Protection against challenge.	(199)
Japanese encephalitis virus (JEV_NSW/22_)	BinJV/JEV_NSW/22_	Mice: Female C57BL/6J and *Ifnar* ^-/-^	2 x 1 µg i.m	No adjuvant	Neutralizing antibody titers. Protection against challenge.	(54)
		Young pigs	(manuscript in preparation)		Antibodies. Protection against challenge.	(55)
West Nile virus (Kunjin)	BinJV/WNV_KUN_	4 month hatchling saltwater crocodiles	2x 10 µg live or UV-C inactivated i.m.	With and without Advax	Neutralizing antibodies. Protection against WNV skin lesions	(52)

Please see **Supplementary Table 2** for details.

Farmed crocodiles in Africa (*Crocodylus niloticus*) and Australia (*C. porosus*) and alligators (*Alligator mississippiensis*) in the USA can be infected by West Nile virus (WNV), with an outbreak of severe neurological disease reported in an alligator farm in Florida in 2002 (49). In Australia, farmed saltwater crocodiles (*C. porosus*) can develop dark spotted “pix” skin lesions, which results in loss of value or rejection of the hides. These lesions arise from infection with Kunjin virus, the Australian strain of WNV, which shows low virulence or is non-pathogenic in humans (50), but affects horses and crocodiles (51, 52). Two intra-muscular (i.m.) vaccinations of hatchling crocodiles with BinJV/WNV_KUN_ vaccine resulted in no detectable skin lesions after WNV_KUN_ challenge (52) (**Table 2**; **Supplementary Table 2**).

An unprecedented outbreak of JEV genotype IV occurred in humans (45 cases, 7 deaths) and pigs (>80 piggeries) in Australia in 2022 (53). Although sera from Imojev (genotype 3) vaccinated humans cross-reacted with a virus isolated from the 2022 outbreak (JEV_NSW/22_), JEV_NSW/22_ was neutralized at significantly lower serum dilutions when compared with genotype 3 JEV isolates (53). This prompted the generation of a BinJV/JEV_NSW/22_ vaccine, which showed efficacy in a mouse model (54), with a media release also reporting a successful trial of this vaccine in pigs (55).

### 2.4 The Aripo virus platform for orthoflavivirus vaccine development

Aripo virus (ARPV) was isolated from *Psorophora albipes* mosquitoes collected in Trinidad in 2008 (56) (**Figure 1**). A chimeric ARPV/ZIKV vaccine was unable to replicate in vertebrate cells (57) and vaccination of C57BL/6J dams resulted in protection of the ~4 week old offspring from ZIKV challenge (58) (**Table 3**; **Supplementary Table 3**). A follow-up study suggested that antibodies played the primary role in protection mediated by the ARPV/ZIKV vaccine, with cell mediated responses playing a minor role (59).

**Table 3:** Aripo virus (ARPV), YN15-283-02 virus and Chaoyang virus chimeric vaccines for pathogenic orthoflaviviruses evaluated in mice.

Pathogenic virus	Chimera	Animal	Dose, route of administration	Adjuvant	Readouts	Ref
Zika virus (DakAr D)	ARPV/ZIKV	Mice: 4 week *IFNα/βR^-/-^*	10^9^ genome copies (GC) s.c.	No adjuvant	Neutralizing antibodies. Protection against challenge	(57)
		Mice: 4 week C57BL/6			Neutralizing antibodies. Protection against challenge. CD8 and CD4 T cell responses.	
		Mice: dams *IFNα/βR^-/-^*			Neutralizing antibodies. Protection against challenge	
		Mice: 4 week C57BL/6J	10^8^ to 10^12^ GC s.c.		Neutralizing antibodies. Protection against challenge (after anti-IFNAR blocking antibody); at 10^12^ GC dose	(58)
		Mice: 4 week C57BL/6J pups	Passive transfer.		Pups from vaccinated dams. Neutralizing antibodies. Protection against challenge (after anti-IFNAR blocking antibody)	
		Mice: *IFNα/βR^-/-^*	Passive transfer		After transfer of serum from vaccinated C57BL/6J mice. Neutralizing antibodies Protection against challenge	(59)
West Nile virus (WNV 3356)	YN15-283-02/WNV	Mice: C57BL/6	3 x 10^6^ FFU i.p.		ELISA antibody titers. Protection against challenge	(61)
Chaoyang virus (CYV)	CYV/ZIKV	Mice: *Ifnar* ^-/-^	10^4^ FFU s.c.		Neutralizing antibodies. Protection against challenge	(62)
		Mice: *Ifnar* ^-/-^	Multiple bites from CYV/ZIKV infected mosquitoes	Not applicable	Neutralizing antibodies. Protection against challenge	(63)

Please see **Supplementary Table 3** for details.

### 2.5 The YN15-283-02 virus platform for orthoflavivirus vaccine development

YN15-283-02 is a mosquito orthoflavivirus that was identified by sequencing the supernatant of C6/36 cells inoculated with a midge homogenate collected in Yunnan, China, with virus recovered using an infectious clone (60). A chimeric YN15-283-02/WNV vaccine was constructed, harvested from the supernatants of infected C6/36 cells, and used to vaccinate C57/BL6 mice. The mice generated Th1 biased antibody responses and were protected from WNV challenge (61) (**Table 3**; **Supplementary Table 3**). YN15-283-02/WNV was unable to replicate in vertebrate cells and was also non-pathogenic in *Ifnar^-/-^* mice (61).

### 2.6 The Chaoyang virus platform for orthoflavivirus vaccine development

Chaoyang virus (CYV) was initially isolated from *Aedes vexans* in China, with a single CYV/ZIKV vaccination providing partial protection from ZIKV challenge (62) (**Table 3**; **Supplementary Table 3**). In addition, immunization of mice via bites from mosquitoes infected with CYV/ZIKV, elicited ZIKV-specific immune responses (increasing after 2 and 3 bites) and conferred protection against ZIKV challenge (63) (**Table 3**; **Supplementary Table 3**). No virus replication was detected in *Ifnar^-/-^* mice bitten by such mosquitoes (63), and no signs of infection were seen in one day old suckling in *Ifnar^-/-^* mice inoculated intracranially with CYV/ZIKV (62).

## 3 Adjuvant issues for ISV-based chimeric virus vaccines

### 3.1 Self-adjuvanting properties of ISV-based chimeric vaccines?

RNA-Seq studies have suggested that ISV-based chimeric vaccines may mediate self-adjuvanting properties via the induction of type I interferon (IFN) responses, triggered via Toll-like receptors (TLRs) and RIG-I (DDX58) (34, 36, 56, 64). TLR and RIG-I agonists, primarily via induction of type I IFNs, are well known to provide adjuvant activity (65–67). Several reports of effective immune response generation without formulation of ISV-based chimeric vaccines with adjuvants (**Tables 1**-**3**; **Supplementary Tables 1**-**3**) would appear to support the contention of self-adjuvanting activity. However, although purification processes have been developed (34, 68), to date these vaccines have not been shown to have been purified to Good Laboratory Practice (GLP) or Good Manufacturing Practice (GMP) standards. Given they are isolated from supernatants of infected insect cell lines, contamination with material from infected cells (69) or cell debris is likely, although cytopathic effects in infected insect cell lines may be moderate rather than overt (70, 71). Contaminants with TLR-stimulating activity might include mRNA and genomic/mitochondrial DNA from the insect cell line, as well as unencapsidated chimeric viral genomic single-stranded RNA (ssRNA), subgenomic RNA, and/or double-stranded RNA (dsRNA) replication intermediates. RNA-Seq data and subsequent bioinformatic analyses would be unable convincingly to distinguish between stimulation of type I IFN stimulated genes (ISGs) via TLR-stimulating contaminates versus true self-adjuvanting activity, such as might be mediated by stimulation of cytoplasmic sensors (like RIG-I). The two pathways induce an extensively overlapping set of ISG mRNAs.

Notwithstanding the aforementioned issues, delivery of ISV-based chimeric vaccine ssRNA into the cytoplasm of vertebrate cells would appear likely, although no clear viral RNA replication has been detected in wild-type vertebrate cells (5, 6, 10, 33, 35, 48, 52). Capping of viral RNA substantially reduces RIG-I stimulation (72). However, as many alphavirus virions can contain uncapped ssRNA genomes (73), incoming viral nucleocapsids containing viral RNA with exposed 5′-triphosphates may trigger RIG-I, without the requirement for viral RNA synthesis/replication (74). Whether a significant proportion of incoming orthoflavivirus chimeric vaccine RNA genomes would be uncapped remains unclear. Other sensors like Protein Kinase R (PKR) and ZAP could also detect cytoplasmic vaccine ssRNA. ZAP binds ssRNA rich in CpG dinucleotides (11, 75) and potently promotes RIG-I signaling (76), with CpGs present with relatively high frequencies in ISVs (10, 77). PKR recognizes short (∼15 bp) stem-loop RNA structures containing flanking ssRNA sequences (78), which are present in alphaviral and orthoflavivirus genomes, both in the untranslated (79–82), and likely also in the translated regions of the genomes (83). PKR has a role in NLRP3 inflammasome activation (84, 85), a pathway also stimulated by aluminium-based adjuvants (86). PKR stimulation also stabilizes IFNβ mRNA, thereby promoting type I IFN responses (87). Also conceivable is stimulation of TLR7, although this would require release of encapsidated vaccine ssRNA from the virion (presumably within or into an endosome), RNA processing, and then access of the products to TLR7 in endosomes (88).

In summary, self-adjuvant activity arising from genomic ssRNA delivered by ISV-based chimeric vaccines can be envisaged. However, this has yet to be formally distinguished from immunostimulatory contaminants by illustrating that purified material produced to GLP/GMP standards, retains adequate self-adjuvanting activity.

### 3.2 Formulation with adjuvants

ISV-based chimeric vaccines provide authentically folded virus particle immunogens to the immune system, much like many established VLP-based vaccines. Most VLP vaccine products currently in the market are adjuvanted (89, 90), and some pre-clinical studies on BinJV-based chimeric vaccines have also used adjuvant formulations (**Table 2**). For instance, formulation of the BinJV/WNV_KUN_ vaccine with Advax slightly improved neutralizing antibody titers in crocodiles, although this did not reach significance (52). A baculovirus manufactured COVID-19 vaccine formulated with Advax, SpikoGen, recently received marketing authorization in Iran (91).

Adjuvants provide dose sparing (less immunogen thus required per dose), with the cost of immunogen manufacture often relatively high for cell culture-derived VLP-based vaccine products (92). Cost considerations are amplified for livestock vaccines, where, despite their clear benefits (93), commercial cost-benefit calculations usually require livestock vaccines to be considerably cheaper than human vaccines. In addition to standard aluminium-based adjuvants (94), new adjuvants for human use and applied to VLP vaccine products (90) have emerged in recent years and include MF59 (95) and the AS0 series (GSK) (e.g. Cervarix, AS04) (96). New adjuvants for livestock products include (i) ImpranFLEX, a proprietary water-based polymer adjuvant used in Ciroflex (Boehringer Ingelheim), a baculovirus-generated VLP product for porcine circovirus type 2, (ii) light liquid paraffin used in Porcilis PCV (Intervet), a baculovirus-generated VLP vaccine for porcine circovirus type 2 (97), (iii) Emulsigen (MVP adjuvants), a new oil-in-water adjuvant that has USDA approval for use in pigs, and (iv) Montanide adjuvants (SEPPIC) approved in the EU.

## 4 Inactivation of ISV-based chimeric virus vaccines?

An ISV-based chimeric vaccine would be viewed as a Genetically Modified Organism (GMO) in most regulatory environments. A series of potential ensuing risks can be perceived after release of these organisms into the environment (see below), with a range of varied restrictions applied in different countries (98, 99). To avoid such risks, ISV-based chimeric vaccines have been inactivated using traditional methods such as formalin fixation (35, 55), UV-irradiation (52) or X-ray irradiation (63). The former clearly reduced induction of neutralizing antibody responses to EILV/CHIKV chimeras, with formalin fixation preventing cell entry (35) and reducing immunogenicity (100) by *inter alia* irreversibly modifying lysine residues [discussed in (101)]. Nevertheless, formalin inactivated ISV-based chimeric vaccines, just like many formalin-inactivated whole virus vaccine products (2), can provide protective immune responses (35, 55). Manufacturing and regulatory processes for formalin inactivation of whole virus vaccines are also well established (102). UV-inactivation of RNA viruses is primarily achieved through uracil and cytosine dimer formation (103). However, irradiation technologies are still primarily in the research and development phase for whole virus vaccines (104, 105) and are not currently used for commercial whole arbovirus vaccine products (2). Delivering the correct irradiation dose safely, evenly and consistently during large scale manufacture (i.e. enough to inactivate, but not too much to damage immunogenicity) represent challenges for these processes. For instance, although a UV-inactivated BinJV/WNV_KUN_ vaccine still afforded protection in mice, neutralizing antibody levels induced by the UV-inactivated vaccine were significantly reduced (106).

Arguably, inactivation of ISV-based chimeric vaccines negates a key advantage of this technology as these vaccines are already intrinsically “inactive”, being unable to replicate in vertebrates. Avoiding the down-stream processes for inactivation and validation of inactivation protocols should represent a key advantage for these ISV-based technologies. Inactivation would remove the GMO classification, and thus the ensuing regulatory hurdles (99), as in most jurisdictions an organism would be defined (in this context) as a replication competent entity. Environmental risks of dissemination and recombination would clearly also be removed by inactivation. However, it should be noted that use of replication competent GMOs as vaccine products is already well established, with a number of licensed live-attenuated arboviral vaccines classed as GMOs e.g. (i) Ixchiq (Valneva), the recently approved CHIKV vaccine that has a 186 nucleotide (62 amino acid) deletion in nsP3, (ii) Imojev (Sanofi), a JEV vaccine, which has the non-structural proteins from YFV 17D, (iii) Dengvaxia (Sanofi), a DENV vaccine, also with a YFV backbone, and (iv) Qdenga (Takeda), where all four serotypes have the non-structural proteins of DENV2. Recombinant virally vectored vaccines are also classified as GMOs with a range of these now also licensed, e.g. (i) Covishield (AstraZeneca) a recombinant adenovirus vaccine for COVID-19, (ii) Raboral (Boehringer Ingelheim), a recombinant vaccinia veterinary vaccine for rabies, and (iii) Trovac-NDV (Boehringer Ingelheim), a recombinant fowlpox livestock vaccine for Newcastle disease (107).

## 5 The role of authentic tertiary and quaternary structures

The importance of presenting the immune system with authentically folded arboviral vaccine immunogens is widely recognized (92, 108), with protective antibodies often targeting quaternary epitopes (109–111). Currently licensed orthoflavivirus and alphavirus vaccines likely deliver antigens with authentically folded tertiary and quaternary protein structures, facilitated by the robust ability of orthoflavi- and alpha-viral structural polyproteins to self-assemble into virion particles in a range of settings (2).

ISV-based chimeric vaccines are fully functional viruses in insect cells and assemble into authentic virion particle structures (33, 48, 61, 112). For instance, low resolution analysis of BinjV chimeras by cryo EM revealed that the virus particles accurately mimic the virion structure of the wild-type pathogens (112). However, subtle differences may emerge to be important, for instance, ortho flavivirus envelope proteins are able to “breath”, adopting more “bumpy” versus “smooth” configurations at the virion surface. Ensuring that an ISV-based chimeric vaccine has the same structure or conformational dynamics as the contemporary pathogenic orthoflavivirus being targeted by the vaccine, may be important for optimizing vaccine efficacy (113).

Authentically folded immunogens are important for induction of antibodies capable of high-affinity and high-avidity binding to the target arbovirus virions and arbovirus infected cells. The vaccine-induced antibodies can be both neutralizing and non-neutralizing, with the latter also mediating a range of protective activities that are generally difficult to measure in clinical trial settings (2). Indirect evidence for a protective role for non-neutralizing, vaccine-induced, antibodies in humans comes from *inter alia* a phase III trial of Qdenga, where neutralizing antibody titers against DENV2, DENV3 and DENV4 did not correlate with protection (114).

## 6 The role of non-structural proteins and CD8 T cell responses

For all the ISV-based chimeric vaccines the non-structural proteins of the pathogenic arboviruses are not present as vaccine antigens. These proteins are nsP1, nsP2, nsP3, nsP4 for alphaviruses, and NS1, NS2A, NS2B, NS3, NS4A, NS4B, NS5 for orthoflaviviruses (**Figure 1**). The ISV versions of these non-structural proteins are synthesized in the insect cell lines used to manufacture the chimeric vaccines. ISV-based chimeric vaccines also contain ssRNA chimeric genomes, which encode the non-structural proteins of the ISV (but are not translated in vaccine recipients) (**Figure 1**).

Ixchiq, the recently approved, live-attenuated CHIKV vaccine, would present CHIKV nsPs to vaccine recipients, whereas this would not occur for non-replicating VLP-based or ISV-based chimeric vaccines. Anti-nsP responses likely do not play a significant role in protection against pathogenic alphavirus infections, a contention supported *inter alia* by successful phase 3 trials recently reported for an aluminum hydroxide adjuvanted, CHIKV VLP vaccine (115). Anti-alphaviral CD8 T cell responses may play a minor role in protection, but they are generally viewed as secondary to antibody responses (116–120). CHIKV appears able to evade surveillance by antiviral CD8 T cells, in part, by nsP2-mediated disruption of MHC-I antigen presentation (121). Nevertheless, induction of CD8 T cells was shown for the EILV/CHIKV vaccine in mice (34) and NHPs (36), and for the ARPV/ZIKV vaccine in mice (59), suggesting that these vaccines can infect and endosome escape, thereby delivering structural protein antigens into the MHC-I processing pathway (122).

In contrast to alphaviruses, protective immune responses directed at orthoflaviviral non-structural proteins, in particular NS1, are well described, with a number of groups pursuing the development of NS1-based vaccines (123–127). Although cross-reactivity of anti-ZIKV NS1 responses with self has been described (128), a causal link to autoimmune disease (129) has yet to be established (130, 131). NS1 proteins of flaviviruses show a degree of sequence homology (132), so live attenuated vaccines such as Dengvaxia (Sanofi) and IMOJEV (Sanofi Pasteur) with a YFV 17D backbone, would likely induce anti-NS1 responses capable of cross-reacting with DENV and JEV NS1 proteins, respectively. Qdenga (Takeda), with a DENV2 backbone, would arguably induce better anti-DENV NS1 responses (2), as sequence homologies between NS1 from DENV2 versus DENV1, 3 and 4 are more substantial (132). However, even if anti-NS1 protein responses can mediate effective protection, a number of licensed orthoflavivirus vaccines achieve adequate protection without inducing such responses, specifically, the inactivated vaccines for (i) JEV, Ixiaro (Valneva), (ii) tick-borne encephalitis virus, Encepur (Bavarian Nordic), and (iii) West Nile virus, Innovator (a horse vaccine from Zoetis). An inactivated virus vaccine for ZIKV was also recently shown to be effective in pregnant marmosets (133).

## 7 Induction of long-term protective immune responses

An ideal characteristic for many alphavirus and orthoflavivirus vaccines would be provision of long-term protective immunity, an issue particularly pertinent for human vaccines in resource poor settings, although clearly less of an issue for short-lived livestock. Perhaps a standout is the YFV 17D vaccine, which provides life-long immunity in humans after a single vaccination (134). Non-replicating, protein-based vaccines are often viewed as providing poor long-term protection. However, this is not entirely accurate with, for instance, the whole-virion-based, formalin inactivated, aluminium hydroxide adjuvanted, hepatitis A vaccine providing protection for >20 years, and the VLP-based, aluminium hydroxide adjuvanted, human papilloma virus vaccine (Gardasil), providing protection for up to 14 years (135). The current COVID-19 mRNA vaccines would appear to perform poorly with respect to durability of protective responses, with neutralizing antibody responses waning within 6 months (136). However, this may be related to an inability to generate spike-specific, long-lived plasma cells (137). The durability of protection after vaccination with mRNA vaccines against arboviruses has yet to be evaluated (138). The same goes for ISV-based chimeric vaccines, and may depend on the choice of adjuvant. Perhaps encouraging, a single dose of a BinJV/ZIKV vaccine, with no adjuvant formulation, provided protective immunity in *Ifnar^-/-^* mice for 15 months (139).

## 8 Vaccine manufacture

Manufacture of human and animal vaccines need to adhere to a series of production and quality control standards, with requirements for human vaccines considerably more onerous than for animal vaccines (140, 141). The number of cell lines available for manufacture of human products is limited, but include CHO (142), HEK293 (143) and Vero cells (144), as well as baculovirus systems that use insect cells from *Spodoptera frugiperda* (armyworm moth) (29, 145). However, to date, no large-scale vaccine production systems have been developed for mosquito cell lines. A desirable property of C6/36 cells is that they do not contain adventitious viruses (146), with such agents having caused problems for human and veterinary vaccine products in the past (147, 148). Another desirable property of ISV-chimeric virus vaccines is that they often grow to higher titers in insect cell lines than their parental viruses (34), with, for instance, the BinJV/ZIKV chimera produced yields of up to ~10^9.5^ CCID_50_/mL or ~7 mg/liter (48). Production and purification of EILV/CHIKV vaccine in C7-10 cells has been described, with culture in serum free medium for 18 h before harvest, followed by Cellufine sulfate column and then sucrose gradient purification (34). This purified vaccine, delivered once i.m. at up to 1.3 x 10^8^ PFU, showed no overt adverse effects in NHPs (36). Manufacturing and purification processes for BinJV chimeric vaccines have also been developed and involve growth in C6/36 cells and purification by density gradient centrifugation (54, 68). A serum free, suspension culture system was recently described for production of BinJV chimeras in C6/36 cells, representing an important step in the development of a scalable manufacturing system (149).

A number of critical steps remain for development of human ISV-based vaccines. These include (i) regulatory clearance for a master cell bank of a suitable mosquito cell line, (ii) characterization of impurities and development of GLP/GMP vaccine production and purification processes, (iii) identification of suitable excipients and storage protocols that preserve vaccine immunogenicity, and (iv) development of quality control processes to monitor vaccine production. Thereafter, phase I human trials, which primarily determine safety, might be initiated. For veterinary vaccines, depending on the animal species, a priority is often cost-effectiveness, so manufacturing costs will generally need to be low (150).

## 9 Safety considerations

### 9.1 Reversion to virulence

Reversion to virulence for ISV-based chimeric vaccines is unlikely as ISV replication is blocked or restricted at various levels in the vertebrate host (4–8). Viral proteins have evolved to interact with various host proteins to promote efficient replication, and to interfere with the hosts anti-viral responses (151–153). These virus-host interactions (or interactomes) are often quite species specific, with, for instance, some ISVs only replicating in a limited number of mosquito species (12, 20, 21). The probability of ISV non-structural proteins undergoing extensive mutations to overcome the multiple restrictions (4–11) and provide an ISV-based chimeric vaccine with the capacity to replicate in vertebrate vaccine recipients with any degree of efficiency, might thus be viewed as extremely low.

### 9.2 Mosquito cell line derived impurities as potential allergens

Mosquitos are found on every continent of the world except Antarctica, with over half the world’s population at risk from diseases spread by mosquitoes (154). Individuals bitten by mosquitoes are exposed to mosquito allergens that are present in the mosquito saliva and injected into the skin by mosquitoes taking a blood meal. Saliva proteins, once injected, generally stimulate a series of acute immune responses (155–158) and induce a local itch response that usually involves IgE-mediated hypersensitivity (159). Local allergic reactions to mosquito bites are usually clinically mild and self-limiting, but in certain individuals can be more severe. For instance, Skeeter syndrome, arising from IgE and IgG responses against mosquito saliva, can occur in immunocompromised individuals, resulting in large local inflammatory reactions, occasionally accompanied by fever and, more rarely, lymphadenopathy (159, 160). However, few, if any, human deaths have occurred as a result of anaphylactic shock caused by a mosquito bite (161). In addition, salivary gland proteins are being considered as potential vaccines for mosquito borne diseases (to induce IgG rather than IgE responses) (162).

C6/36 and C7-10 cells are derived from *Aedes albopictus* larvae, and likely share allergens (or contain immunologically cross-reactive allergens) with mosquito saliva proteins, as supernatants from C6/36 cultures injected into human skin can induce hypersensitivity and anaphylactic reactions (163). Unfortunately, no C6/36 allergens doses (i.e. µg of protein per injection) were provided in this study. The EILV/CHIKV vaccine, purified by Cellufine sulfate affinity chromatography column (AMS-BIO) and sucrose gradient centrifugation (34), was shown to cause little or no skin hypersensitivity reactions in mice or guinea pigs sensitized to mosquito bites (34, 36). The level of purification and thus the allergen dose is likely to be a central issue. For instance, pre-existing egg allergy is no longer considered a contra-indication for modern egg-derived influenza vaccines (164). This is not the case for the yellow fever vaccine, which contains higher levels of egg allergens (165). Future research will likely need to characterize the mosquito-derived proteins that co-purify with the ISV-based vaccines, and compare these with allergenic mosquito salivary proteins (166) in order to better understand the potential risks. Nevertheless, predisposition to Skeeter syndrome (and perhaps other anaphylactic conditions) may emerge to be a contra-indication for C6/36 and C7-10-derived vaccines.

### 9.3 Transmission to mosquitoes

As determined for other live attenuated arboviral vaccines, like the YFV 17D vaccine (167, 168) and the VEEV vaccine (TC-83) (169), the capacity of ISV-based chimeric vaccines to be transmitted to, and by, mosquitoes may need to be characterized. Concerns might arise from (i) introduction of a GMO into the environment and (ii) the potential of the vaccine to interact with the mosquito virome (see below), although both ISV and pathogenic arbovirus genes are already present in the environment. Another potential concern is transmission of a GMO to other humans without their consent; however, given the small doses delivered by mosquito bites and the absence of replication post-delivery, human welfare is unlikely to be impacted.

As ISV-based chimeric vaccines do not replicate in the vaccine recipient, the mosquito would need to be infected by virus from the vaccine inoculum. The mosquito would thus need to take a blood meal at or near the injection site and close to the time of inoculation. The mosquito proboscis penetrates about 2-3 mm into the skin to reach a blood capillary, whereas subcutaneous (and certainly intramuscular) injections are usually deeper, with dissemination into local capillaries likely to be transient. The titers imbibed by the mosquito would also need to be high enough to initiate infection and transmission, with a number of threshold barriers to infection and transmission recognized for mosquitoes. These barriers primarily regulate virus escape from the midgut, entry into the salivary glands, replication in the salivary glands, and release of virus into the saliva (170–172). CYV/ZIKV was able to infect and disseminate to the saliva in *Aedes aegypti*, and less so in *Aedes albopictus*, after artificial blood meals containing 10^8^ FFU/mL (63). Once initiated, amplification of ISV-based chimeric vaccines in the mosquito might be expected, given the high titer replication of ISV-based chimeric viruses in insect cell lines (35, 48). Formulation of the ISV-based chimeric vaccines with adjuvants, for instance, by adsorption onto aluminium-based adjuvants or by formulation in emulsion adjuvants, would likely reduce the probability of uptake and infection of mosquitoes.

### 9.4 Recombination

The ability of alphaviruses to recombine is well described (173, 174), whereas orthoflaviviruses appear to have a much lower propensity to recombine (175, 176). Recombination would require the ISV-based chimeric vaccine and another virus to replicate in the same cell, something which would likely only be possible after infection of mosquitoes. Superinfection exclusion may mitigate against ISV-based chimeric vaccine and another virus infecting the same cell in the mosquito (2, 177). Nevertheless, any potential interactions with complex mosquito viromes might be viewed as unpredictable, given our limited understanding of these complex ecosystems (12, 13, 178). However, ISVs and pathogenic arboviruses have already extensively co-infected mosquitoes, with all the genes in ISV-based chimeric vaccines thus having already been present in mosquito populations over evolutionary time periods.

## 10 Needle-free delivery opportunities

Needle-free vaccine delivery overcomes needle-phobia, eliminates needle-stick injuries and the necessity for sharps disposal, and may emerge to be attractive and cost-effective in mass vaccination campaigns, especially in resource poor settings (179). Two devices have been licensed for delivery of vaccines, Stratis (PharmaJet) for delivery of inactivated influenza vaccine (Afluria, Seqirus) in the USA, and Tropis (PharmaJet) for delivery of the ZyCoV-D DNA COVID-19 vaccine (Zydus Lifesciences) in India (180) for *Restricted Use in Emergency Situations*. These devices are applied to the skin and deliver vaccines via a narrow precise fluid stream that penetrates the skin. Similar needle free systems have also been developed for the livestock industry and have the advantage of reducing pain, saving time, avoiding lesions in muscles (meat products), as well as avoiding the aforementioned safety issues associated with needle use (181).

An alternative needle-free vaccine delivery technology involves microarray patches or microprojection arrays (182), which involves dry coating of vaccine onto microprojections arrayed on small patches that are applied to the skin and that deliver antigen to skin antigen presenting cells (183, 184). A number of microarray patches have been evaluated in phase I human trials for *inter alia* influenza, measles, rubella, and SARS-CoV-2 vaccines. They were generally regarded as safe and well tolerated, inducing similar or increased immune responses when compared with conventional needle-based vaccination (185–188).

The technology developed by Vaxxas (188–190) has been used to deliver a BinJV-based chimeric vaccine for DENV that provided protection in a mouse model (68, 191).

## 11 Conclusions

A number of mosquito ISV-based chimeric vaccines for human arboviral pathogens have been developed and been shown to generate protective immunity in preclinical studies in mice and NHPs. However, human vaccine applications will require a certified mosquito cell line and GMP manufacturing and purification processes to be established, with evaluation of different adjuvant formulations also likely required. Ultimately, ISV-based chimeric vaccines may have to compete with mRNA vaccine technologies (192, 193) on *inter alia* cost of goods, durability of responses, and/or safety. Manufacture of ISV-based vaccines is at an early stage and data is currently limited. Conceivably, the manufacturing performance of mosquito cell line systems might emerge to be comparable with baculovirus systems (145); however, cell line-derived, whole-virus vaccines that need inactivation and adjuvanting generally have a relatively high cost of goods (194). Future developments in mRNA vaccine technology may also reduce the costs of this new technology (195). Specific adjuvants may be required to promote responses and durability of protection of ISV-based chimeric vaccines in humans (2, 135). New developments may also lead to improvements in the durability of responses after vaccination with mRNA vaccines (196). The safety profile of mRNA vaccines is now well established with billions of doses delivered globally (197). The safety profile for ISV-based vaccines has yet to be formally established and must await phase I human trials.

Livestock applications have been illustrated for BinJV/WNV_KUN_ for crocodiles and BinJV/JEV for pigs. Although unable to replicate in vaccine recipients, inactivation (e.g. formalin) of ISV-based chimeric vaccines removes the regulatory hurdles associated with release of GMOs and avoids any risk of transmission to mosquitoes. Whether such vaccines would emerge to be cost-effective enough for livestock markets has yet to be determined.
