# Supplementary material for: Insect-specific virus platforms for arbovirus vaccine development
Source: Front Immunol. 2025 Mar 14;16:1521104. doi: 10.3389/fimmu.2025.1521104 (PMC11949993; doi:10.3389/fimmu.2025.1521104)
Supplement: Supplementary file 1 [file DataSheet1.pdf]

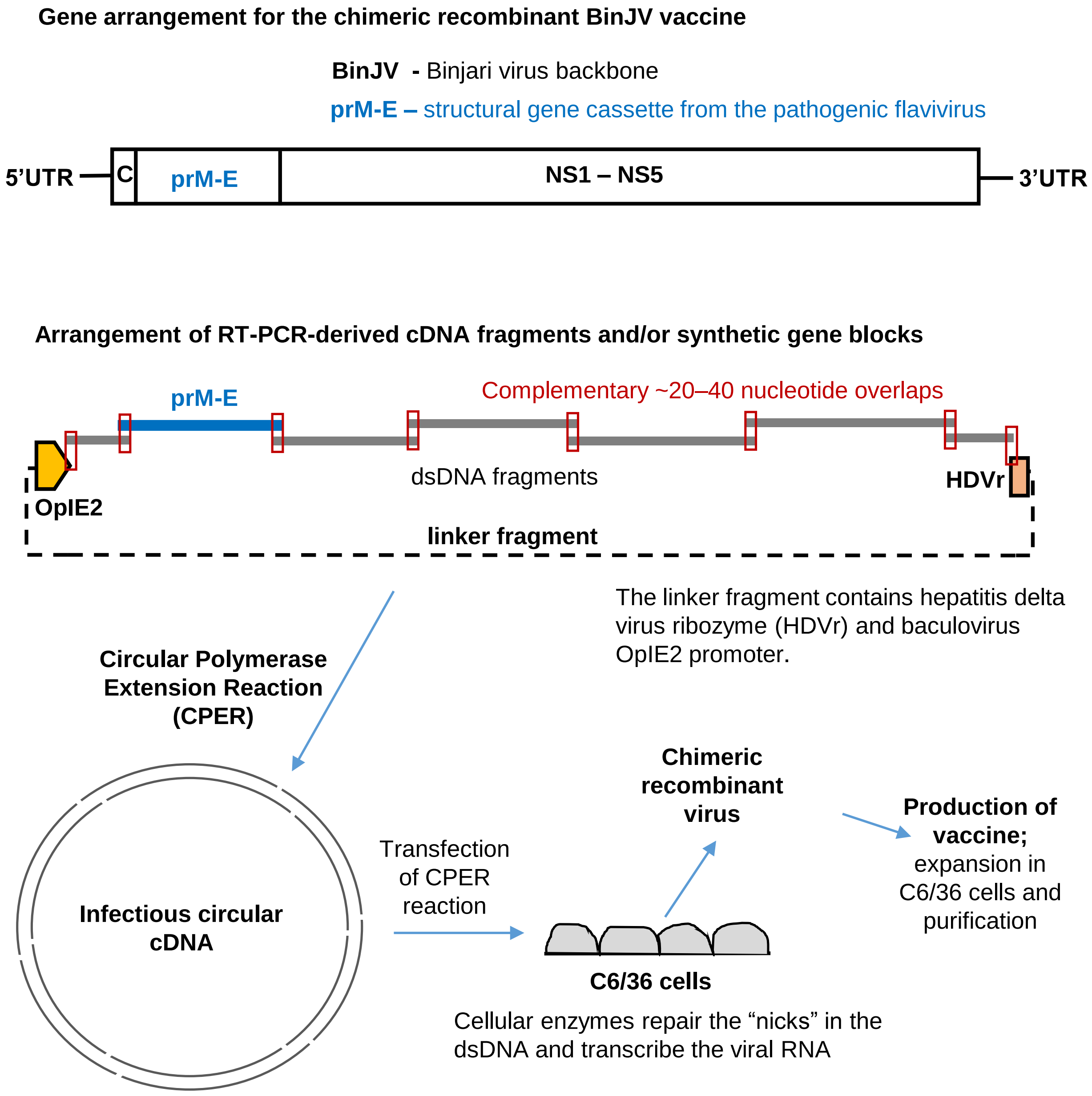

Supplementary Figure 1. Schematic illustrating the use of CPER to generate a chimeric BinJV vaccine. dsDNA fragments covering the BinJV genome with the exception of prM-E genes are amplified by RT-PCR from BinJV RNA. The prM-E gene cassette from a pathogenic vertebrate-infecting flavivirus are either RT-PCR amplified from viral RNA or purchased as a synthetic gene block. The linker fragment contains an OpIE2 promoter and hepatitis delta virus ribozyme (HDVr) linked by a spacer region to facilitate circularisation. All fragments contain complementary overlapping ends (red boxes). Fragments are assembled into circular dsDNA by CPER with high fidelity DNA polymerase. The reaction is then directly transfected into C6/36 cells to produce the chimeric recombinant virus. The technique can be used for ISV chimeras (Hobson-Peters et al 2019), as well as a range of other viruses (Amarilla et al 2021). YYV was reconstructed by CPER using gene blocks as the virus was identified by metagenomic sequencing (and has – as yet – not been isolated from mosquitoes) (Bell et al 2024).
