# Supplementary material for: Insect-specific virus platforms for arbovirus vaccine development
Source: Front Immunol. 2025 Mar 14;16:1521104. doi: 10.3389/fimmu.2025.1521104 (PMC11949993; doi:10.3389/fimmu.2025.1521104)
Supplement: Supplementary file 2 [file DataSheet2.pdf]

Supplementary Table 1

Details for Table 1. Eilat virus (EILV) chimeric vaccines for pathogenic alphaviruses evaluated in mice and NHPs.

Chimera	Animal	Dose, route of administration	Details of vaccine induced responses and vaccine-mediated protection	Ref
EILV/CHIKV	Mice: young C57BL/6	8.8 log_{10}PFU live or formalin-inactivated s.c.	-Neutralizing antibodies; $PRNT_{80}$ day 28, means of ≈1 in 500 vs. ≈1 in 100 for inactivated EILV/CHIKV.<br>-IFNγ+CD8+ T cells; means of ≈35 vs. ≈15 $x10^4$ for inactivated.<br>-Protection from challenge; significant reduction in mean foot swelling days 3-12 post challenge.	(1)
	Mice: adult *Ifnar*$^{-/-}$	8.5 log_{10}PFU live or formalin-inactivated s.c.	-Neutralizing antibodies; $PRNT_{80}$ day 28, means of ≈1 in 1000 vs. ≈1 in 160 for inactivated EILV/CHIKV.<br>-Protection against challenge; 100% protection against lethal challenge and significant protection against weight loss.	
	Mice: C57BL/6	10^8 PFU i.p	-IgM and IgG antibodies; ELISA assays - day 14 mean ODs ≈0.4 for IgM and ≈0.6 for IgG vs. ≈0.1 background (differences significant).<br>-Antibody secreting cells; mean of ≈500/10^7 cells (background at ≈100/10^7).<br>-IFNγ+CD8+ T cells; peak day 28 means of ≈45 $x10^4$, background ≈25 $x10^4$ (differences significant).	(2)
	NHPs (*Macaca fascicularis*)	1.3 x 10^6 or 1.3 x 10^8 PFU i.m.	-Neutralizing titers; mean $PRNT_{50}$ ≈2-2.5 (log10).<br>- IgG+ B cells /10^6 cells; mean of ≈40 for each protein (C, E3, E2 and E1).<br>- IFNγ ELISPOT; means of ≈10/10^6 cells for each protein (C, E3, E2 and E1).<br>- Protection; adoptive transfer of serum to mice given a lethal infection – significantly delayed mortality and 20% vs. 0% survival.	(3)
EILV/VEEV	Mice: CD-1	10^8 PFU s.c.	Neutralizing antibodies; mean $PRNT_{80}$ ≈1 in 1000 at day 6.<br>Protection; 100% survival after s.c. challenge	(4)
EILV/EEEV	Mice: CD-1	10^8 PFU s.c.	Neutralizing antibodies; mean $PRNT_{80}$ ≈1 in 320 at day 28.<br>Protection; 100% survival after s.c. challenge	

Supplementary Table 2

Details for Table 2. Binjari virus (BinJV) chimeric vaccines for pathogenic orthoflaviviruses evaluated in mice, crocodiles and pigs.

Chimera	Animal	Dose, route of administration	Details of vaccine induced responses and vaccine-mediated protection	Ref
BinJV/ZIKV	Mice: Male *Ifnar*$^{-/-}$	2 µg and 20 µg i.m.	Without Adjuvant<br>-Total IgG ELISA titer at 5 weeks mean at 2 ug dose means ≈1 in 10^3-10^4, at 20 ug ≈1 in 2 x10^4 (backgrounds below detection).<br>- 50% neutralizing antibody titers at 5 weeks means at 2 ug dose ≈1 in 10^2 (1/6 non-responders), and at 20 ug ≈2 in 2x10^2 (5/5 responders), (backgrounds below detection).<br>-Protection; no detectable viremia and no testes damage at both doses post ZIKV challenge. No significant weight loss at 20 ug dose.<br><br>Plus Addavax<br>-Total IgG ELISA titer at 5 weeks, means at 2 ug dose ≈1 in 10^3, at 20 ug ≈1 in 10^4 (backgrounds below detection).<br>- 50% neutralizing antibody titers at 5 weeks means at 2 ug dose ≈1 in 10^2, and at 20 ug ≈1 in 10^2, (backgrounds below detection).<br>-Protection; no detectable viremia and no testes damage at both doses post ZIKV challenge. No significant weight loss at 20 ug dose.	(5)
	Mice: Female *Ifnar*$^{-/-}$	2x 10 µg and 20 µg i.m.	Without adjuvant<br>-Total IgG ELISA titers at 8 weeks means at 2x10 ug dose ≈1 in 10^5, at 20 ug ≈1 in 5 x10^4 (backgrounds below detection). No non responders.<br>-50% neutralizing antibody titers at 8 weeks means at 2x10 ug dose ≈1 in 10^3, at 20 ug ≈1 in 10^3 (backgrounds below detection).<br>-Protection; no detectable viremia or weight loss for both regimens post ZIKV challenge.<br><br>Plus Addavax<br>-Total IgG ELISA titers at 5 weeks means at 20 ug dose ≈1 in 10^5 (backgrounds below detection). No non responders.<br>-50% neutralizing antibody titers at 5 weeks means at 20 ug dose ≈1 in 10^3 (backgrounds below detection).<br>-Protection; no detectable viremia post ZIKV challenge. Some weight loss in Addavax group.	
	Mice Pregnant *Ifnar*$^{-/-}$	1x 10 µg i.m.	-Total IgG ELISA titers at 5 weeks means at 10 ug dose ≈1 in 10^5 (backgrounds below detection). No non responders.<br>-50% neutralizing antibody titers at 5 weeks means at 10 ug dose ≈1 in 3x10^2 (backgrounds below detection).<br>-Protection; no detectable viremia post ZIKV challenge in dams. No ZIKV RNA detected in placentas or fetal tissues at E12.5.	(6)
	Mice: Female *Ifnar*$^{-/-}$	1x 20 µg i.m.	-50% neutralizing antibody titers at 14 months means at 20 ug dose ≈1 in 50 with and without Addavax (backgrounds below detection).<br>-Protection; no detectable viremia, significant reduction in weight loss, and 100% survival post ZIKV challenge with and without Addavax.	(7)

BinJV/WNV_{KUN}	Mice: Male and female CD1	Live or UV inactivated, 1x and 2x 1 µg and 5 µg s.c.	-Endpoint ELISA titers; at 8 weeks ranges from means of ≈1 in 500 (for to 1x 1 ug) to ≈1 in 20,000 (for 2x 1ug + adjuvant). At 5 ug doses ranges from means of ≈1 in 1600 (for to 1x 5 ug) to ≈1 in 20,000 (for 2x 5 ug + adjuvant).<br>-Neutralizing antibody; at 8 weeks ranges from means of ≈1 in 100 (for to 1x 1 ug) to ≈1 in 2000 (for 2x 1ug + adjuvant). At 5 ug doses ranges from means of ≈1 in 400 (for to 1x 5 ug) to ≈1 in 2000 (for 2x 5 ug + adjuvant).<br>Responses were significantly lower after UV treatment of the vaccine.<br>-Protection against WNV_{NY99} challenge; 100% survival vs. 40% survival in controls, viremia below detection, reduced weight loss and near complete prevention of disease and mortality for all formulations and doses.	(8)
BinJV/DENV2	Mice: Female AG129	2x micro-array patch.	After 2 doses of high density microarray patch vaccination.<br>-ELISA titers; IgG mean log t_{50} of ≈4 (significantly higher than i.d. vaccination with 1 ug)<br>-Neutralizing antibody; mean log t_{50} of ≈2.5 (significantly better than i.d. vaccination with 1 ug).<br>-Protection against challenge; 100% survival vs 0% for controls, >1 log reduction in viremia, up to 1 log reduction in serum NS1 levels.	(9)
BinJV/YFV_{17D}	Mice: Female *Ifnar*$^{-/-}$	2x 5 µg, 10 µg or 20 µg of i.m.	Plus MPLA/QS-21 adjuvant<br>-50% neutralizing antibody titers at week 10, means at all doses ≈1 in 500 (backgrounds below detection).<br>-Protection; no detectable viremia, no significant weight loss, and reductions in H&E detectable liver lesions.	(10)
BinJV/$JEV_{NSW/22}$	Mice: Female C57BL/6J and *Ifnar*$^{-/-}$	2 x 1 µg i.m	-Neutralizing antibody titers; means at ≈1 in 500 in both strains (backgrounds below detection).<br>-Protection against challenge; viremia undetectable in both mouse strains, inhibition of weight loss in *Ifnar*$^{-/-}$mice, and spleen and brain titers reduced to below detection in *Ifnar*$^{-/-}$ mice.	(11)
	Young pigs		Details of antibody responses and protection against challenge are not yet available (manuscript in preparation).	(12)
BinJV/WNV_{KUN}	4 month hatchling saltwater crocodiles	2x 10 µg live or UV-C inactivated i.m.	-Neutralizing antibodies; at 8 week mean sof ≈1 in 500 end point neutralization assay for live vaccine plus adjuvant (Advax), ≈1 in 200 live no adjuvant, UV inactivated ≈1 in 60, UV inactivated plus adjuvant ≈1 in 100.<br>-Protection against WNV skin lesions from a mean of 22 lesions for PBS controls to no detectable lesions in all vaccinated groups.	(13)

Supplementary Table 3

Details for Table 3. Aripo virus (ARPV), YN15-283-02 virus and Chaoyang virus chimeric vaccines for pathogenic orthoflaviviruses evaluated in mice

Chimera	Animal	Dose, route of administration	Details of vaccine induced responses and vaccine-mediated protection	Ref
ARPV/ZIKV	Mice: 4 week *IFNα/βR*$^{-/-}$	10^9 genome copies (GC) s.c.	-Neutralizing antibodies; $PRNT_{80}$ week 4, mean of ≈1 in 500.<br>-Protection against challenge; no detectable viremia, no significant weight loss, compete protection from mortality.	(14)
	Mice: 4 week C57BL/6		-Neutralizing antibodies; $PRNT_{80}$ week 4, mean of ≈1 in 250.<br>-Protection; no detectable viremia, no significant weight loss.<br>- CD8+IFNγ+ day 35 mean ≈0.4% of splenocytes that were CD45+ cells (background ≈0.2), Th1 ≈0.65% (background ≈0.35%), Th2 ≈0.5% (background ≈0.24%). Also significant secretion of IL-2, IFNγ and TNF after *in vitro* stimulation.	
	Mice: dams *IFNα/βR*$^{-/-}$		-Neutralizing antibodies; $PRNT_{80}$ day 29, mean of ≈1 in 300.<br>-Protection against ZIKV challenge of pregnant dams; no detectable viremia or tissue titers in dams, no significant weight loss or brain virus titers in neonates.	
	Mice: 4 week C57BL/6J	10^{12} GC s.c.	-Neutralizing antibodies; $PRNT_{50}$ 4 weeks, mean of ≈1 in 3500.<br>-Protection; ZIKV challenged after treatment with anti-IfnaR monoclonal antibody (MAR1-5A3), no detectable viremia. (Repeat expt in 6 week mice also showed no significant weight loss).	(15)
	Mice: 4 week C57BL/6J pups	Passive transfer	Four week old pups borne to vaccinated dams.<br>-Neutralizing antibodies in pups; $PRNT_{50}$ 4 weeks, mean of ≈1 in 2600.<br>-Protection in pups; limited weight loss, no mortality and no detectable viremia; ZIKV challenge after MAR1-5A3 treatment.	
	Mice: *IFNα/βR*$^{-/-}$	Passive transfer	After high-dose passive transfer of serum from vaccinated C57BL/6J mice<br>-Neutralizing antibodies; $PRNT_{50}$ mean of ≈1 in 450.<br>-Protection; significant reduction in mortality, weight loss and viremia.<br>Also showed that T cells play a minor role in protection.	(16)
YN15-283-02/WNV	Mice: C57BL/6	3 x 10^6 FFU i.p.	-ELISA antibody means titers IgG1 ≈1 in 2800, IgG2c ≈1 in 3500 day 28.<br>-Protection; significant reduction in weight loss, no detectable viremia and 100% survival.	(17)
CYV/ZIKV	Mice: *Ifnar*$^{-/-}$	10^4 FFU s.c.	-Neutralizing antibodies; 50% neutralizing antibody titer day 28 mean of ≈1 in 180.<br>-Protection; reduction in serum viral RNA, 100% survival and significant reduction in body weight loss. No detectable viral RNA in brain.	(18)
	Mice: *Ifnar*$^{-/-}$	Multiple bites from CYV/ZIKV infected mosquitoes	-Neutralizing antibodies; FRNT50 week 5 ≈1 in 200 to 1 in 800 depending on ZIKV strain.<br>-Protection; reduction in serum viral RNA, 100% survival and significant reduction in body weight loss. Also show transmission blocking activity.	(19)

References

1. Erasmus JH, Auguste AJ, Kaelber JT, Luo H, Rossi SL, Fenton K, et al. A chikungunya fever vaccine utilizing an insect-specific virus platform. *Nat Med* (2017) 23(2):192-9. doi: 10.1038/nm.4253.
2. Adam A, Luo H, Osman SR, Wang B, Roundy CM, Auguste AJ, et al. Optimized production and immunogenicity of an insect virus-based chikungunya virus candidate vaccine in cell culture and animal models. *Emerg Microbes Infect* (2021) 10(1):305-16. doi: 10.1080/22221751.2021.1886598.
3. Adam A, Woolsey C, Lu H, Plante K, Wallace SM, Rodriguez L, et al. A safe insect-based Chikungunya fever vaccine affords rapid and durable protection in cynomolgus macaques. *npj Vaccines* (2024) 9:251. doi: 10.1101/2024.05.21.595029.
4. Erasmus JH, Seymour RL, Kaelber JT, Kim DY, Leal G, Sherman MB, et al. Novel Insect-Specific Eilat Virus-Based Chimeric Vaccine Candidates Provide Durable, Mono- and Multivalent, Single-Dose Protection against Lethal Alphavirus Challenge. *J Virol* (2018) 92(4):10-1128. doi: 10.1128/JVI.01274-17.
5. Hobson-Peters J, Harrison JJ, Watterson D, Hazlewood JE, Vet LJ, Newton ND, et al. A recombinant platform for flavivirus vaccines and diagnostics using chimeras of a new insect-specific virus. *Sci Transl Med* (2019) 11(522):eaax7888. doi: 10.1126/scitranslmed.aax7888.
6. Hazlewood JE, Rawle DJ, Tang B, Yan K, Vet LJ, Nakayama E, et al. A Zika Vaccine Generated Using the Chimeric Insect-Specific Binjari Virus Platform Protects against Fetal Brain Infection in Pregnant Mice. *Vaccines (Basel)* (2020) 8(3):496. doi: 10.3390/vaccines8030496.
7. Hazlewood JE, Tang B, Yan K, Rawle DJ, Harrison JJ, Hall RA, et al. The Chimeric Binjari-Zika Vaccine Provides Long-Term Protection against ZIKA Virus Challenge. *Vaccines (Basel)* (2022) 10(1):85. doi: 10.3390/vaccines10010085.
8. Vet LJ, Setoh YX, Amarilla AA, Habarugira G, Suen WW, Newton ND, et al. Protective Efficacy of a Chimeric Insect-Specific Flavivirus Vaccine against West Nile Virus. *Vaccines* (2020) 8(2):258. doi: 10.3390/vaccines8020258.
9. Choo JJY, Vet LJ, McMillan CLD, Harrison JJ, Scott CAP, Depelsenaire ACI, et al. A chimeric dengue virus vaccine candidate delivered by high density microarray patches protects against infection in mice. *npj Vaccines* (2021) 6(1):66. doi: 10.1038/s41541-021-00328-1.
10. Yan K, Vet LJ, Tang B, Hobson-Peters J, Rawle DJ, Le TT, et al. A Yellow Fever Virus 17D Infection and Disease Mouse Model Used to Evaluate a Chimeric Binjari-Yellow Fever Virus Vaccine. *Vaccines (Basel)* (2020) 8(3):368. doi: 10.3390/vaccines8030368.
11. Harrison JJ, Nguyen W, Morgan MS, Tang B, Habarugira G, de Malmanche H, et al. A chimeric vaccine derived from Australian genotype IV Japanese encephalitis virus protects mice from lethal challenge. *NPJ Vaccines* (2024) 9(1):134. doi: 10.1038/s41541-024-00903-2.
12. UQ_News. Vaccine to protect pigs from Japanese encephalitis virus. https://www.uq.edu.au/news/article/2023/02/vaccine-protect-pigs-japanese-encephalitis-virus. Accessed Oct 2024.
13. Habarugira G, Harrison JJ, Moran J, Suen WW, Colmant AMG, Hobson-Peters J, et al. A chimeric vaccine protects farmed saltwater crocodiles from West Nile virus-induced skin lesions. *npj Vaccines* (2023) 8(1):93. doi: 10.1038/s41541-023-00688-w.
14. Porier DL, Wilson SN, Auguste DI, Leber A, Coutermarsh-Ott S, Allen IC, et al. Enemy of my enemy: a novel insect-specific flavivirus offers a promising platform for a Zika virus vaccine. *Vaccines* (2021) 9(10):1142.
15. Tanelus M, López K, Smith S, Muller JA, Porier DL, Auguste DI, et al. Exploring the immunogenicity of an insect-specific virus vectored Zika vaccine candidate. *Scientific Reports* (2023) 13(1):19948. doi: 10.1038/s41598-023-47086-9.
16. Porier DL, Adam A, Kang L, Michalak P, Tupik J, Santos MA, et al. Humoral and T-cell-mediated responses to an insect-specific flavivirus-based Zika virus vaccine candidate. *PLOS Pathogens* (2024) 20(10):e1012566. doi: 10.1371/journal.ppat.1012566.
17. Zhang HQ, Li N, Zhang ZR, Deng CL, Xia H, Ye HQ, et al. A Chimeric Classical Insect-Specific Flavivirus Provides Complete Protection Against West Nile Virus Lethal Challenge in Mice. *J Infect Dis* (2024) 229(1):43-53. doi: 10.1093/infdis/jiad238.
18. Dong H-L, Chen Z-L, He M-J, Cui J-Z, Cheng H, Wang Q-Y, et al. The Chimeric Chaoyang-Zika Vaccine Candidate Is Safe and Protective in Mice. *Vaccines* (2024) 12(2):215.

19. Wen D, Ding LS, Zhang Y, Li X, Zhang X, Yuan F, et al. Suppression of flavivirus transmission from animal hosts to mosquitoes with a mosquito-delivered vaccine. *Nature Communications* (2022) 13(1):7780. doi: 10.1038/s41467-022-35407-x.
